# Mental Health in Construction Industry: A Global Review

**DOI:** 10.3390/ijerph22050802

**Published:** 2025-05-20

**Authors:** Apurva Pamidimukkala, Sharareh Kermanshachi, Deema Nabeel Almaskati

**Affiliations:** 1 Department of Civil Engineering, University of Texas at Arlington, Arlington, TX 76019, USA; apurva.pamidimukkala@mavs.uta.edu (A.P.); deemanabeel.almaskati@mavs.uta.edu (D.N.A.); 2 Industrial, Manufacturing, and Systems Engineering, University of Texas at Arlington, Arlington, TX 76019, USA

**Keywords:** mental health, construction workforce, risk factors, strategies, occupational stress

## Abstract

Work-related stress is a major contributing factor to the relatively high number of deaths from suicide and other mental disorders among those who work in the construction industry. Despite the knowledge that unmanaged stress can manifest as depression and ultimately trigger thoughts of suicide or even the act itself, workers’ mental health is often neglected. This study aims to identify the risk factors that are at the root of the workers’ mental health challenges, as well as the strategies that can be initiated to overcome or at least mitigate them. To accomplish this, a systematic literature review was conducted using the PRISMA method, and 132 relevant publications that met the pre-defined inclusion criteria were selected for further analysis. From the data analysis, 45 risk factors were identified and classified into five categories based on the literature and the definition of stressors. The most frequently cited mental health risk factors were revealed to be gender inequality (diversity and equity category), poor working conditions (health-related category), work overload (job demand category), poor work–life balance (organizational category), and lack of social support (personal category). Thirty-two (32) intervention strategies were identified and divided into primary, secondary, and tertiary types. It was evident from the findings that combining all three types of interventions is the most effective way to improve the mental health of the construction workforce. The findings from this study provide valuable insights for policymakers and regulatory agencies who develop and implement policies aimed at improving mental health and occupational safety in the construction industry.

## 1. Introduction

The construction industry is globally recognized as a hazardous, challenging, and stressful work environment that negatively impacts its workers’ mental health [1,2]. Construction workers face numerous stresses that negatively impact their mental and physical well-being, including the inherent dangers of the work, the need to maintain high levels of productivity and make complicated decisions, and the expectation that they will conform to the industry’s perception of toughness [3]. An Australian survey by MATES in Construction in 2018 indicates that about one in five individuals in the construction workforce suffers from a diagnosed psychological illness within any given year. Approximately 42% of construction workers in the United Kingdom report having experienced occupational stress, anxiety, and depression, which they attribute to their work environment [4]. Research conducted in Canada revealed that 83% of workers in the construction sector have encountered mental health issues of varying magnitudes [5], and another study revealed that 16% of construction workers in the United States exhibit considerable mental discomfort [6]. The COVID-19 pandemic further exacerbated mental health challenges within the construction workforce, highlighting increased stress, anxiety, and workload pressures due to disruptions, job uncertainty, and changing work conditions [7,8]. Overall, it is evident that the mental health of individuals in the construction industry is a significant issue with serious repercussions in multiple countries.

The construction industry has elevated rates of suicide globally, attributable to the presence of psychological hazards in the work environment [9]. For example, in Australia, one in six fatalities in the construction industry is attributed to suicide [9]. In the United States, suicide rates among inexperienced construction workers are 4.25 times higher than the national average [10]. These countries also have the highest death rate from opioids [11,12], which are prescribed for managing pain from work-related musculoskeletal injuries [13], but whose long-term use all too frequently leads to overdoses, suicides, and depression [11]. Other prevalent mental health concerns in the construction industry include anxiety disorders, alcohol use, sleep difficulties, and post-traumatic stress disorder [14,15,16].

The alarming statistics on the poor mental health and high suicide rates of construction workers have motivated researchers worldwide to investigate the factors contributing to these issues [17]. Frimpong et al. [2] determined the frequency of physical and mental health conditions affecting Ghana’s youth in the construction industry, and Sun et al. [18] and Golzad et al. [19] identified the factors that contribute to mental health disorders. Despite these studies and others conducted during the past two decades on this topic, however, the current body of literature lacks sufficient data that explores and analyzes the risk factors.

To fill this research gap, a comprehensive review was performed of the existing literature to find answers to the following research questions: (1) What factors affect the mental health of a construction workforce? (2) How are the factors ranked? (3) What strategies can reduce the consequences of the risk factors? This study will raise awareness of the mental health issues and specific challenges that construction workers, employers, and stakeholders face in the workplace and will facilitate open dialogue and collaboration for effectively addressing them. The findings of the study will provide valuable insights for policymakers and regulatory agencies who develop and implement policies aimed at improving mental health and occupational safety in the construction industry.

The contributions of this study are multifaceted. The key risk factors for mental health challenges within the construction workforce are identified, categorized, and ranked, and this provides valuable insight into those that require immediate attention. Intervention strategies for mitigating the consequences of the risk factors are also identified, with a focus on the type of prevention methods and stratified approaches tailored to different organizational levels. These findings provide a practical framework for improving mental health within the construction industry and impart guidelines to employers, policymakers, and mental health professionals for implementing targeted, evidence-based interventions that can improve the well-being of the workforce and enable the tailoring of policies in other high-risk sectors.

## 2. Methodology

**Figure 1:**
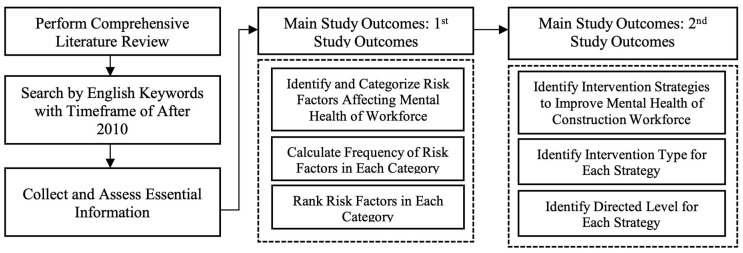
Research framework.

Figure 1 presents the research design developed to achieve the objectives of this study. A systematic literature review was conducted, using the PRISMA methodology, to identify the existing literature on the topic. Scopus, PubMed, and Google Scholar databases were used to retrieve the articles. The initial search was conducted in February 2024, and this was updated in November 2024. Keywords such as “mental health”, “risk factors”, “psychological hazards”, “strategies”, “construction industry”, “workforce”, “suicide”, etc., were employed using different combinations. The inclusion criteria comprised the following: English language publications from 2010 to 2024, concentrating on mental health issues within the construction industry, and addressing risk factors and strategies. The search was also limited to articles published from the year 2010, as there was a heightened awareness of the necessity to enhance mental health within the construction industry after this period.

**Figure 2:**
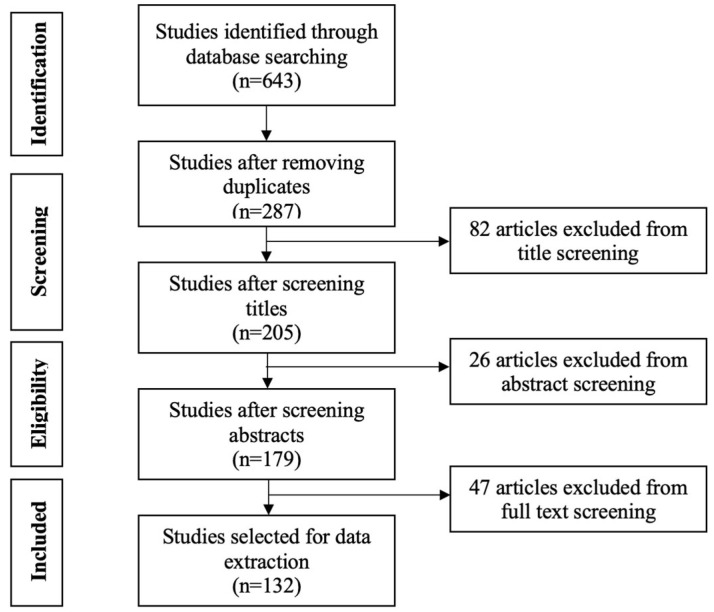
PRISMA screening process.

A preliminary search of databases yielded a total of 643 publications. Next, the collected database was further reviewed to identify duplications, resulting in a total of 287 publications. The articles were then filtered based on their title and abstract, and the full texts were reviewed to ensure their relevancy to the objectives of this study. The preliminary screening of titles disregarded 82 publications that were not pertinent to the research topic. Next, a comprehensive review of abstracts was carried out, resulting in a further 26 articles being removed. Lastly, full-text screening facilitated the selection of publications that closely aligned with the established inclusion criteria. This step removed 47 articles, and the remaining 132 studies revealed the risk factors that contribute most significantly to mental health problems in construction personnel and facilitated a discussion of strategies. Figure 2 presents the PRISMA screening process adopted in this study.

## 3. Results and Discussion

### 3.1. Identifying and Classifying the Risk Factors That Affect the Mental Health of Construction Workforce

Identifying the underlying causes of mental health problems is the first step toward alleviating them, and in the construction arena, this means exploring the reasons for work-related stress. Construction workers are subject to excessive demands to finish projects by the established deadline within the designated budget while adhering to industry and project standards [18]. The importance of their contributions to society cannot be overstated, yet the completion of projects often takes priority over their mental health [17]. It is, therefore, imperative that those in management positions fully comprehend the factors that adversely affect their employees’ mental health so that they can help them and cultivate a work environment that prioritizes their mental well-being.

The identified risk factors were categorized into five groups to consolidate similar stressors: diversity and equity, health-related, job demand, organizational, and personal. The classification was conducted according to studies that identified several of these stressors [17,20] and the definitions of the stressors. The factors were also ranked within each category according to how often they are mentioned in the existing literature. This increased our understanding of the risk factors and will help scholars and project managers protect the mental health of their employees. The categories and rankings are presented below.

#### 3.1.1. Diversity and Equity Risk Factors (DR)

Table 1 shows that gender inequality and sexual harassment are the most significant risk factors affecting female construction workers. The literature reveals that females are often paid less than their male colleagues [21,22] and experience many types of harassment, including unwanted physical contact [23,24] and sexual, verbal, and physical assault. Women are also afforded fewer opportunities for advancement than their male counterparts [22] and, therefore, experience higher levels of anxiety and despair. The studies also indicate that male coworkers frequently refuse to comply with a female supervisor’s instructions, which diminishes their sense of authority and results in their feeling less supported by management [25].

Language barriers are another significant cause of mental health problems among construction workers, as the inability to effectively communicate with supervisors, colleagues, and/or clients can create a sense of inadequacy, increase stress levels, and lead to errors, accidents, or conflicts that further exacerbate feelings of anxiety and insecurity [26,27].

**Table 1:** Diversity and Equity Risk Factors.

ID	Risk Factor	Description	Source	Frequency	Rank
DR1	Gender inequality	Men have more opportunities for advancement and are treated more fairly than women.	[21,22]	29	1
DR2	Sexual Harassment	Female workers experience many types of harassment, including sexual, verbal, and physical assault.	[23,24]	25	2
DR3	Limited job opportunities for women	Limited job opportunities hinder career growth for female construction workers.	[22,28]	22	3
DR4	Language barriers	Language barriers prevent effective communication with supervisors and colleagues.	[26,27]	15	4
DR5	Racial discrimination	Racial discrimination is prevalent on construction sites.	[5,16]	9	5
DR6	Age discrimination	Younger workers face significant challenges in construction workplaces.	[2,29]	5	6
DR7	Cultural value conflicts	Conflicts arise from differences in cultural values.	[1,26]	2	7

DR refers to diversity and equity risk factors.

#### 3.1.2. Health-Related Risk Factors (HR)

Table 2 reflects the literature’s focus on the role that environment plays in employee mental health. Inadequate ergonomic designs, insufficient lighting, a lack of cleanliness, and other physical deficiencies are frequently cited as factors that significantly impact mental health, as the discomfort and stress they cause decrease employee productivity, thereby creating even more stress. Ergonomic desk and seating shortcomings can lead to physical strain and discomfort that exacerbates mental stress, insufficient lighting not only affects visibility but also dampens moods and energy levels [2,5], and unclean or cluttered spaces can evoke feelings of unease and lack of control.

Ill-fitting personal protective equipment (PPE) was cited 19 times in the existing literature and was ranked second among the health-related risk factors affecting the mental health of a construction workforce. (See Table 2) The discomfort, physical strain, and increased risk of injury that it causes incite feelings of frustration, helplessness, and anxiety [25,28].

Occupational injuries ranked third among the health-related risk factors, as injuries such as accidents, falls, cuts, and other forms of physical harm can profoundly impact mental health [30] by triggering feelings of fear, anxiety, and vulnerability. Trauma associated with workplace injuries may lead to post-traumatic stress disorder (PTSD), depression, or heightened stress levels that affect both work performance and personal well-being [31,32], and fear of recurrence or the inability to return to work due to injuries can further exacerbate the challenges.

**Table 2:** Health-Related Risk Factors.

ID	Risk Factor	Description	Source	Frequency	Rank
HR1	Poor working environment	Environments that are not ergonomically designed, lack adequate lighting, are not clean, or have other physical deficiencies	[2,5]	24	1
HR2	Ill-fitting PPE	PPE that does not fit properly and has the potential to cause discomfort, physical strain, and injury	[25,28]	21	2
HR3	Occupational injuries	Injuries that occur as a direct result of job-related activities, including accidents, falls, cuts, and other physical harm	[30,32]	18	3
HR4	Musculoskeletal pain	Muscle, nerve, tendon, joint, or spinal disc injuries or pain caused by repetitive strain or overuse	[33,34]	11	4
HR5	Personal traumas	Emotional or psychological injuries resulting from accidents, violence, or loss, which affect an individual’s mental health	[8,35]	5	5
HR6	Poor medical services	Lack of adequate medical services	[5,29]	2	6

HR refers to health-related risk factors.

#### 3.1.3. Job-Demand Risk Factors (JR)

Table 3 provides a list of the job-demand risk factors, a description of each, and how they are ranked, based on the number of times they were cited in the literature. Work overload, which is defined as the disparity between the requirements of a project and a worker’s ability to meet them [36,37], is the top-ranked risk factor in the literature. Rigid time constraints and the complexity of construction projects are major components of the heavy workloads that negatively impact construction employees’ work–life balance and are likely to lead to even higher levels of stress.

Role ambiguity, ranked as the second most contributing risk factor to mental health problems, arises from a lack of information and a clear definition of the tasks that have to be performed for a project [27]. This is particularly a problem in large construction projects with tight deadlines that involve multiple trade contractors, and it can result in employee burnout as well as work overload [38].

Role conflict, ranked third among all the identified job demand factors, occurs when workers are faced with incompatible and inconsistent expectations from two or more parties that they are unable to meet simultaneously [38,39]. The chaos resulting from this type of conflict compromises employee productivity and causes undue stress.

Interpersonal conflicts related to the complexity and uncertainty of projects, as well as the involvement of a substantial number of stakeholders [19], were cited 20 times in the studied literature. Timely and effective conflict management is vital to the mental well-being of those involved and the outcome of the project [40].

Work underloads are also instrumental in undermining employees’ mental health, as the lack of meaningful tasks may cause them to feel disengaged and undervalued [41,42], induce feelings of boredom and apathy, decrease motivation, and ultimately contribute to psychological distress and poor mental health. Over time, prolonged periods of work underload can increase the risk of depression, anxiety, and burnout among workers.

#### 3.1.4. Organizational Risk Factors (OR)

Table 4 provides a list, description, and ranking of organizational risk factors and shows that a poor work–life balance is the most common culprit of compromised mental health among construction workers. The long hours, irregular schedules, extended time away from family, and lack of time for personal pursuits [47,48] that are inherent in many construction projects make it difficult for workers to achieve a healthy balance between their work and personal lives. The lack of time to relax and engage in enjoyable pursuits often induces anxiety, sadness, burnout, chronic stress, exhaustion, and feelings of isolation [49].

Low job control, demonstrated by an inability to participate in decision making, enforcement of rigid work schedules and strict rules, and authoritarian work cultures [33], was mentioned as a major organizational risk factor 33 times in the reviewed literature, making it the second most highly cited organizational risk factor [20].

A work environment that does not promote career development was cited 27 times in the literature, making it the third-ranked organizational risk factor. Without opportunities for career advancement, skill development, and professional growth, construction workers feel stuck in dead-end jobs and experience feelings of stagnation, frustration, and disillusionment that exacerbate stress and anxiety [29,50]. A lack of career development support can also result in decreased job satisfaction and compromised overall well-being, as workers may be more susceptible to burnout and struggle to see their future in the industry [51].

Inadequate support from supervisors, colleagues, and/or the organization negatively affects workers’ mental health, as it promotes feelings of isolation and unimportance [26]. An unsupportive work environment can exacerbate stress, anxiety, and feelings of being overwhelmed, as workers struggle to cope with job demands and navigate challenges on their own [30]. Without access to resources, guidance, and encouragement from their peers and superiors, construction workers may experience heightened levels of job dissatisfaction and emotional distress that affect their overall health and job performance [52,53].

Procedural injustice, such as unfair decision making, was cited 19 times in the literature as a risk factor for mental health issues [36,54]. When construction workers feel that they are being treated unfairly or inconsistently in matters such as promotions, assignments, or disciplinary actions, it erodes their confidence in the fairness and integrity of the workplace and initiates feelings of anger, resentment, and mistrust; low morale, and minimal employee engagement [44].

**Table 4:** List of Organizational Risk Factors.

ID	Risk Factor	Description	Source	Frequency	Rank
OR1	Poor work-life balance	An excessive amount of time and effort is devoted to professional responsibilities at the expense of personal commitments	[47,48]	41	1
OR2	Low job control	Lack of decision-making opportunities	[20,33]	33	2
OR3	Lack of environment that promotes career development	Environment that lacks opportunities and/or support for career development and promotions	[29,50]	27	3
OR4	Low job support	Lack of sufficient support from supervisors and colleagues	[30,52]	25	4
OR5	Procedural prejudice	Inequitable decision-making processes	[36,54]	19	5
OR6	Lack of recognition	Inadequate rewards or recognition of employee accomplishments	[55,56]	14	6
OR7	Job insecurity	Lack of job stability	[41,49]	11	7
OR8	Inadequate freedom of expression	Limited opportunities to express thoughts, opinions, or ideas freely and openly	[50,57]	7	8
OR9	Lack of training	Lack of job training	[17,25]	4	9
OR10	Lack of feedback	Lack of feedback on improving performance	[11,58]	3	10
OR11	Poor communication	Lack of or unclear communication among project teams	[40,59]	2	11
OR12	Lack of human resources	Shortage of project team members/workers	[8,60]	2	12

OR refers to organizational risk factors.

#### 3.1.5. Personal Factors (PR)

Table 5 presents a list of the personal risk factors that impact construction workers’ mental health. Personal risk factors are specific pressures or challenges in an individual’s personal life that cause stress and can negatively impact their mental and emotional well-being. Lack of social support significantly impacts the mental health of construction workers by increasing stress levels, fostering feelings of isolation and loneliness, and heightening the risk of mental health disorders such as depression and anxiety. Without emotional support and practical assistance from a network of family, friends, and colleagues, workers may struggle to cope with the demanding and often hazardous nature of their jobs [5,61], making them vulnerable to decreased job satisfaction, lower productivity, and the adoption of poor coping mechanisms, such as substance abuse.

Type A behavior, a personality trait characterized by a high level of competitiveness, impatience, aggression, and a constant sense of urgency [29], is the second most cited personal risk factor in the literature. Type A construction workers may experience heightened stress and mental health challenges from the demands and pressures of their jobs, and their relentless drive and impatience can lead to burnout, strained relationships with their colleagues, and an increased risk of stress-related health issues [27,62].

### 3.2. Strategies to Improve the Mental Health of a Construction Workforce

Table 6 provides a list of the strategies (S) cited in the literature for improving the mental health of construction workers. The identified strategies not only address existing mental health issues but also prevent potential problems by mitigating some of the risk factors, such as work overload, lack of support, poor work–life balance, etc. These strategies aid in reducing the likelihood of mental health issues and provide support to those affected individuals. Employers are responsible for ensuring a healthy workplace environment that fosters positive health and well-being, and the high incidence of mental health issues in the construction sector can be alleviated by implementing preventative initiatives that foster a culture conducive to seeking assistance [29]. Many construction workers refrain from asking for assistance because of the shame and stigma associated with mental health [65,66]. Increased suicide rates have been noted among male construction workers, a phenomenon that Campbell and Gunning [67] hypothesize is linked to men’s reluctance to seek assistance due to concerns that doing so undermines their masculinity. To address this problem, it is recommended that self-compassion training be conducted to alleviate the shame linked with mental health issues [48] and to assist individuals with managing work-related stress. These training methods can be employed alongside improved access to mental health treatments and other readily available resources, such as online and telehealth options, to mitigate the stigma associated with mental health and assist individuals in managing work-related stress [11].

The strategies were evaluated based on the type of intervention and directed levels, and it was determined that to be effective, they should incorporate primary, secondary, and tertiary interventions. Primary interventions seek to reduce stress by offering workplace training opportunities (S4), secondary interventions focus on assisting employees in managing stressors by providing constructive feedback (S12), and tertiary interventions encompass providing counseling services (S11) [68]. The literature reveals that investing in primary and secondary prevention is generally considered cost-effective in the long term, as it reduces the need for more expensive tertiary interventions [29].

**Table 6:** Strategies to Improve Mental Health of Construction Workforce.

ID	Strategies	Risk Factors	Prevention Type	Directed Level	Source
S1	Develop policies to eliminate harassment and bullying.	DR2	Primary	I, O	[60]
S2	Implement policies to promote equality regardless of gender, age, and race.	DR1, DR6, DR7, DR8	Primary	I, O	[30]
S3	Provide resources for assistance in coping with stressors such as financial, marital, and family issues.	PR6, PR7	Tertiary	I	[18]
S4	Offer workplace training opportunities.	OR7, OR9	Secondary	O	[39]
S5	Promote prompt resolution of workplace conflicts.	JR4	Secondary	O	[54]
S6	Implement measures to enhance cooperation between supervisors and subordinates.	OR4, PR3	Primary	I, O	[69]
S7	Foster strong workplace relationships.	OR4	Primary	I, O	[70]
S8	Redesign tasks to reduce interdependency.	JR7	Secondary	I	[71]
S9	Streamline tasks and shifts.	JR1	Primary	I	[26]
S10	Hire more personnel to reduce individual workloads.	JR1, OR12	Primary	O	[20]
S11	Provide counseling or other tools for managing mental health problems.	OR7	Tertiary	O	[68]
S12	Provide workers with constructive feedback.	OR10, PR9	Secondary	I	[46]
S13	Provide a supportive physical working environment.	HR1, HR2	Primary	O	[72]
S14	Provide opportunities for employees to express their opinions and participate in decision-making.	OR2, OR8	Secondary	I	[66]
S15	Provide opportunities for growth and promotion at work.	DR3	Primary	I	[4]
S16	Provide opportunities for workers to utilize their abilities and skills.	JR5	Primary	I	[73]
S17	Provide clear instructions, information, and work objectives.	JR2, JR3	Primary	O	[9]
S18	Implement a fair decision-making process.	OR5	Primary	O	[10]
S19	Provide adequate materials and equipment for performing assigned tasks.	DR4	Primary	O	[26]
S20	Promote cordial relationships with coworkers.	PR1, PR5	Primary	I, O	[69]
S21	Offer fair and adequate compensation.	PR6	Primary	O	[10]
S22	Provide opportunities for rewards and recognition.	OR6, PR9	Primary	O	[53]
S23	Foster a flexible work environment.	OR1	Primary	I, O	[74]
S24	Encourage open and transparent communication between team members.	OR11	Primary	I, O	[75]
S25	Implement better recruitment strategies.	DR1	Primary	O	[56]
S26	Increase opportunities for career development.	OR3	Primary	O	[42]
S27	Encourage task prioritization.	JR6	Primary	I, O	[70]
S28	Offer resources and support for managing stress.	JR8	Secondary	O	[43]
S29	Implement a thorough contract review process.	JR10	Primary	O	[76]
S30	Provide training on effective communication skills.	JR9, DR5	Primary	O	[77]
S31	Offer access to employee assistance programs.	PR2, PR8	Secondary	O	[78]
S32	Offer education and awareness programs.	PR4	Primary	O	[5,79]

S refers to strategies, I refers to individual level, and O refers to organizational level.

## 4. Implications

The findings of this study offer several important guidelines that will help industry practitioners and policymakers foster a healthier, more supportive work environment. First, classifying the risk factors into categories such as diversity and equity, health-related issues, and job demands highlights the need for interventions that are tailored to specific mental health problems. For instance, addressing gender inequality and sexual harassment among female workers can lead to the development of targeted anti-harassment training that creates a safer and more inclusive work environment for them. Likewise, improving communication and reducing language barriers can foster a sense of inclusion and reduce stress related to miscommunication. Mental health initiatives such as stress management workshops and access to counseling services will demonstrate construction companies’ support of their workers and reduce the adverse mental health impacts associated with these risk factors.

This study revealed that poor working conditions, including inadequate ergonomics, insufficient lighting, and unclean workspaces, significantly contribute to mental health stress. Addressing these health-related risk factors by providing better lighting, ergonomic workstations, and a clean work environment will alleviate mental stress as well as reduce physical discomfort. Providing appropriately fitting PPE was also highlighted as an essential strategy, as ill-fitting PPE causes physical strain, discomfort, and anxiety. Construction companies should therefore prioritize investments in high-quality, well-designed PPE to reduce mental health strain.

Work overload, role ambiguity, and role conflict were identified as significant job-demand risk factors, and to mitigate these issues, construction companies should implement strategies like clear role definitions, workload management tools, and realistic project deadlines. Ensuring that job roles are explicitly described and progress is regularly monitored will help reduce role ambiguity and prevent burnout caused by unclear expectations. Additionally, scheduling breaks and setting realistic timeframes for project completion can help alleviate stress from work overload.

The identification of interpersonal conflicts, often exacerbated by the complexity of large construction projects and the involvement of multiple stakeholders, highlights the need for effective conflict resolution strategies. Construction companies should implement formal conflict resolution training for supervisors and workers to enhance communication and reduce tensions. Encouraging regular team meetings, providing mediation services, and fostering an open-door policy for reporting grievances can prevent conflicts from escalating and mitigate their impact on workers’ mental health. Promoting a culture of mutual respect and collaboration among workers can also help reduce stress by eliminating misunderstandings and disagreements.

This study’s findings suggest that policymakers and regulatory bodies should integrate mental health strategies into occupational health and safety guidelines to protect workers’ mental and physical well-being. These strategies might include regular mental health assessments, stress management programs, and establishing regulations for ergonomic and environmental safety standards.

The findings of this study form a foundation for the development of predictive risk assessment tools. By incorporating the identified mental health risk factors into ongoing monitoring systems, construction companies can detect early signs of stress and intervene proactively. Regular mental health screenings, surveys, and stress audits can be used to identify workers at risk of mental health issues and facilitate early intervention through counseling or adjustments to workloads. The development of predictive models based on these factors will help companies identify and manage mental health risks more effectively, ensuring that workers receive timely support before stress leads to more severe psychological issues.

## 5. Conclusions

This study explores the risk factors that significantly and negatively impact the mental health of construction workers and proposes a comprehensive set of strategies that overcome the mental health risks. From the literature, 45 risk factors were identified and classified into five categories: diversity and equity, health-related factors, job demands, organizational, and personal. Gender inequality, poor working conditions, work overload, poor work–life balance, and a lack of social support were the most frequently cited risk factors causing mental issues such as anxiety, depression, etc.

The 32 strategies that were identified offer a robust framework for addressing construction workers’ mental health challenges. These were categorized into primary, secondary, and tertiary prevention measures and targeted both the organizational and individual levels. By implementing these strategies, industry stakeholders can create a safer, more supportive work environment that promotes mental well-being and enhances overall productivity. The study’s findings emphasize the importance of proactive measures and the need for ongoing support to address mental health issues effectively.

The findings of this study will be highly beneficial to stakeholders in the construction industry. Employers can use the insights to develop targeted interventions that address key mental health challenges and facilitate a more productive and satisfied workforce. Employees will benefit from improved workplace practices and support systems that enhance their overall well-being. Policymakers can craft informed regulations to protect workers’ mental health, and mental health professionals can tailor their services to the unique needs of construction workers, fostering a healthier, more equitable, and efficient industry.

Despite the comprehensive nature of this study, it is limited by the scope of the identified risk factors, which may not encompass all the stressors that construction workers may experience. Additionally, the findings are based on the available literature, which may predominantly reflect certain regions or cultural contexts and impact the generalizability of the results. The feasibility of implementing the proposed strategies also presents a challenge, as factors such as the availability of resources, the organizational culture, and workforce acceptance present possible roadblocks. Furthermore, future research may benefit from a targeted examination of how these differences might interact with the risk factors identified in this study.

## Figures and Tables

**Table 3:** List of Job-Demand Risk Factors.

ID	Risk Factor	Description	Source	Frequency	Rank
JR1	Work overload	Heavy workload demands that require working under pressure at a rapid pace for extended periods of time	[5,36]	32	1
JR2	Role ambiguity	Ill-defined job duties	[27,38]	27	2
JR3	Role conflict	Assignment of incompatible tasks	[38,39]	25	3
JR4	Interpersonal conflict	Tensions and disagreements between employees in the workplace	[19,40]	20	4
JR5	Work underload	Underutilization of skills, boring and repetitive work	[41,42]	16	5
JR6	Task interdependency	Two or more tasks that depend on one another to complete a goal	[2,43]	13	6
JR7	Cognitive demands	Work demands that require high levels of cognitive vigilance and alertness	[44,45]	7	7
JR8	Emotional demands	Work demands that require dealing with people in different interpersonal contexts	[44,46]	4	8
JR9	Client demand	Clients’ expectations and requirements pertaining to project costs and schedules	[36]	1	9
JR10	Contract pressure	Stress induced by contractual obligations	[36]	1	10

JR refers to health-related risk factors.

**Table 5:** List of Personal Risk Factors.

ID	Risk Factor	Description	Source	Frequency	Rank
PR1	Lack of social support	Poor social interaction with co-workers, friends, and family	[5,61]	22	1
PR2	Type A behavior	Type A behavior is manifested in competitive, aggressive, and time-driven actions.	[29,62]	18	2
PR3	Problem(s) with superior	Poor relationship with supervisor	[12,63]	13	3
PR4	Alcohol and drug use	Substance use caused by job-related physical illnesses and/or stress	[64]	11	4
PR5	Social isolation	The feeling of being alone or disconnected from others	[31,59]	6	5
PR6	Financial insecurity	Fears regarding inadequate income	[35,37]	4	6
PR7	Marital Status	Stress based on relationship problems	[16,30]	4	7
PR8	Family conflicts	Disputes or strained relationships with family	[35,43]	3	8
PR9	Loss of control	The degree to which individuals perceive the relationship between their personal actions and outcomes	[27,54]	2	9
PR10	Fear of failure	Fear of experiencing negative outcomes	[51,55]	2	10

PR refers to personal risk factors.

## Data Availability

No datasets were generated or analyzed during the current study.
